# Sensors and Robotics for Digital Agriculture

**DOI:** 10.3390/s23167255

**Published:** 2023-08-18

**Authors:** Aristotelis C. Tagarakis, Dionysis Bochtis

**Affiliations:** Institute for Bio-Economy and Agri-Technology (iBO), Centre for Research and Technology-Hellas (CERTH), 6th km Charilaou-Thermi Rd., 57001 Thessaloniki, Greece; d.bochtis@certh.gr

The latest advances in innovative sensing and data technologies have led to an increasing implementation of autonomous systems in agricultural production processes. Physical autonomous systems can accomplish even more complex tasks, while cyber ones can support timely, accurate and informed decision making, leading to more efficient farm management and improved profitability in the context of precision agriculture and Agriculture 4.0. This combination has transformed agricultural robots to machines with embedded awareness [1,2] also capable of interacting with other machines [3,4,5], as well as with human labor [6,7,8]. On the other hand, sensing technologies in agriculture continuously provide a vast amount of data necessary for the development and implementation of AI technologies [9,10,11].

All the above constitutes a closed-loop interaction between the disciplines of sensing, AI, and robotics technologies (Figure 1). This interaction is the basis of the present Topical Collection under the purpose of highlighting the corresponding advancements in the domain of precision agriculture. In this collection, a total of 10 articles are included covering different aspects of this interaction approach, including topics such as agri-robotics awareness, human–robot interaction, AI in crop and livestock production, and digital twins in the context of modern agriculture. 

With the recent advances in sensing methods and data acquisition technologies in agriculture, a vast amount of data became available, paving the way to exploring the utilization of artificial intelligence in agriculture. This is the main topic that is analyzed in the comprehensive review provided by Benos et al. [12], as an update of the previous review presented in [9]. Machine learning refers to a subset of artificial intelligence and has considerable potential for handling numerous challenges in the establishment of knowledge-based farming systems. In this study, a thorough review of recent literature on machine learning in crop, water, soil, and livestock management is analyzed. Maize, wheat, cattle, and sheep were the most investigated crops and animals, respectively. This study is anticipated to constitute a guide of the potential advantages of machine learning in agriculture.

Regarding machine learning applications in crop management, two works are presented in the collection. Farkhani et al. [13] propose the use of a multi-layer attention procedure to present an interpretation of the Deep Neural Network’s (DNN) decisions through a high-resolution attention map for the classification of weeds. The proposed architecture can improve the resolution and location of weed areas for efficient weed management. The second work deals with the mapping of agricultural environments. The study presented by Anagnostis et al. [14] proposes an approach for the segmentation of trees in commercial orchards using aerial images obtained by unmanned aerial vehicles (UAVs). The methodology is based on a deep learning convolutional neural network and was proven to be efficient for the automated detection and localization of tree canopies. The trained model was tested on never-before-seen orthomosaic images, achieving performance levels up to 99%, demonstrating the robustness of the proposed approach.

Deep learning was also utilized in livestock management for the detection and tracking of pigs for the quantification of social contacts as described in Wutke et al. [15]. A convolutional neural network (CNN) was applied on video footage to detect the location and orientation of pigs tracking the animals’ movement trajectories over a given period using a Kalman filter (KF) algorithm. This enabled the automatic identification of social contacts in the form of head–head and head–tail contacts. The specific study demonstrated the effectiveness of the methodology to enhance animal monitoring systems. The second study in this collection concerning technological and digital advances in livestock regards the use of neck-mounted collars equipped with accelerometers for monitoring and classifying dairy cattle behaviors (Pavlovic et al.) [16]. Such sensor systems automatically provide information about key cattle behaviors, such as level of restlessness and ruminating and eating time, assessing the overall welfare, at animal level. Within this work, the development of algorithms for the classification of cattle states is described. The results showed that the classification model that was based on linear discriminant analysis using features selected through Backward Feature Elimination provided the most balanced tradeoff between performance and computational complexity. 

The second group of works regard applications of robotics in agriculture. Crop harvesting is one of the most demanding, time-consuming, and labor-intensive operations in high-value crops such as fruit and nut trees, grapes, and various vegetable crops. Due to its seasonal character, securing the work force to address this task is a great challenge [17]. Therefore, a lot of effort has been invested in the development of autonomous or semi-autonomous crop-harvesting systems. In most cases, due to the complexity of the operation, intelligent systems are needed [18]. A review conducted by Navas et al. [19] has been included in the current collection that addresses the task of automatic crop harvesting, focusing on the specifications of soft grippers. Soft robotics and soft grippers are promising approaches in this field due to the specifications required to meet hygiene and manipulation requirements in unstructured environments when working with delicate products. This review provides an insight into soft end-effectors for agricultural applications, emphasizing robotic harvesting, aiming to serve as a guide in the design of soft grippers for fruit harvesting robots in soft robotics for Agriculture 4.0.

Apart from harvesting, there is a plethora of other field management activities that are laborious and time-consuming and are subjected to automation. In the study presented by Kitic et al. [20] an Autonomous Robotic System was developed for real-time, in-field soil sampling and analysis of nitrates in the soil. The system combines a set of modules including a commercial robotic platform, an anchoring module, a sampling module, a sample preparation module, a sample analysis module, and a communication module. The procedure starts with the definition of the sampling locations using a dedicated cloud-based platform which processes satellite images using artificial intelligence. Then, automated soil sampling takes place; each sample is analyzed on the spot and georeferenced, providing a map which can be used for precision-based fertilizing.

The situational awareness and navigation of autonomous robotic platforms in agricultural fields is a particularly challenging and demanding task due to the irregular nature and the complexity of such environments. Therefore, mapping the environment for targeted robotic applications in agricultural fields is challenging due to the high spatial and temporal variability which make these environments highly unpredictable [21]. The aim of the study presented by Tagarakis et al. [22] was to investigate the use of consumer-grade RGB-D (red, green, blue and depth) and unmanned aerial (UAV) and ground vehicles (UGV) for autonomous mapping of the environment in commercial orchards and for providing structural information of the trees such as height and canopy volume. Two systems were used; the ground-based system consisting of a UGV with an RGB-D camera and the aerial-based system which consisted of a UAV equipped with high accuracy RTK-GPS and a precise imaging system. The results from the ground-based mapping system were compared with the three-dimensional (3D) orthomosaics acquired by the UAV. Both systems performed adequately well. The fusion of the two datasets (from the ground and above) provided the most precise representation of the trees. In the pursuit of optimizing the efficiency, flexibility, and adaptability of agricultural operations, digitalization and automatization of agricultural practices are considered as the means to achieving the goals of agricultural production and addressing its modern challenges. However, unmanned systems, aerial or ground, show autonomy at some level and interact with other dynamic elements in the fields such as agricultural machinery and humans. Consequently, a new sector has emerged focused on human–robot interaction (HRI) in agriculture. A systematic review of the advances in the interaction between humans and agricultural robots was conducted by Benos et al. [23], reviewing the scholarly literature to capture the current progress and trends in this promising field while identifying future research directions. Based on the findings of the review, there is a growing interest in the specific research field which combines perspectives from several disciplines. In terms of crops, melons, grapes, and strawberries were the ones with the highest interest for HRI applications, mainly due to their high value perspective and the low availability of traditional machinery automations due to the nature of these cropping systems. Collaboration and cooperation were the most preferred interaction modes, with various levels of automation being examined in the cited studies. Due to the complexity of the agricultural environments and the tasks taking place in agricultural operations, there is still a long way to go towards the establishment of viable, functional, and safe human–robot systems [24,25]. 

As already mentioned, the digitalization of agriculture is the way forward to the future of farming in the framework of Agriculture 4.0, improving production systems and addressing food security, climate protection, and resource management. Due to the complexity and dynamic nature of agricultural production, sophisticated management systems are required supporting farmers and farm managers in making informed and improved decisions. In the review presented by Nasirahmadi et al. [26], the concept of utilizing digital twins and digital technologies and techniques is presented. A digital twin is the virtual representation of a physical system. In agriculture, this can be regarded as the virtual representation of a farm, providing the potential for enhancing productivity and efficiency while minimizing energy usage and losses. A general framework of digital twins in soil, irrigation, robotics, farm machinery, and food post-harvest processing in agriculture is provided. 

To conclude, the current Topical Collection provides insights into advanced ICT systems applied in precision agriculture and digital farming steering towards Agriculture 4.0. The collection includes works that cover multi-disciplinary applications in both crop and livestock production systems. The outcomes from the reported articles highlight the importance of digital systems, sensing technologies, and advanced data analysis methodologies for making informed decisions supporting the sustainability of future farming.

## Figures and Tables

**Figure 1:**
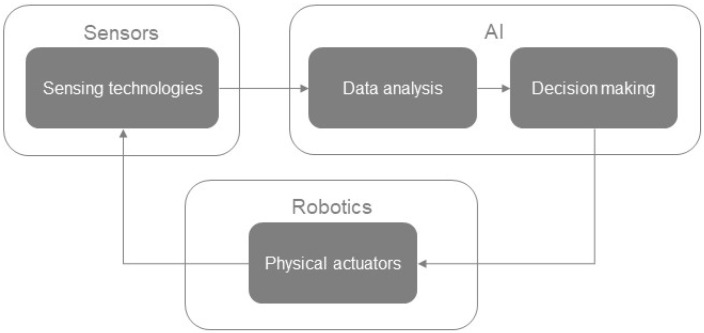
The closed-loop approach for the interaction of the sensors, AI, and robotics entities.
